# Designing and Simulation Assessment of a Chair Attachment Air Blowing Methods to Enhance the Safety of Prolonged Sitting

**DOI:** 10.3390/biomimetics8020194

**Published:** 2023-05-08

**Authors:** Mahmoud Z. Mistarihi, Ammar A. Al-Omari, Abdullah F. Al-Dwairi

**Affiliations:** 1 Department of Industrial Engineering, Hijjawi Faculty for Engineering Technology, Yarmouk University, Irbid 21163, Jordan; 2 Department of Mechanical and Industrial Engineering, Faculty of Engineering, Liwa College of Technology, Abu Dhabi P.O. Box 41009, United Arab Emirates; 3 Department of Industrial Engineering, College of Engineering, Jordan University of Science and Technology, Al-Ramtha 3030, Jordan

**Keywords:** excessive sitting, musculoskeletal disorders, ergonomics, engineering characteristics

## Abstract

Musculoskeletal disorders and the stagnation of sitting are among the side effects of excessive sitting in awkward sitting positions. In this study, a developed chair attachment cushion design with an optimal air blowing technique is proposed to eliminate the negative side effects of prolonged sitting. Instantaneously reducing the contact area between the chair and its occupant is the fundamental goal of the proposed design. The fuzzy multi-criteria decision-making approaches represented by FAHP and FTOPSIS were integrated to evaluate and select the optimal proposed design. An ergonomic and biomechanics assessment of the occupant’s seating position while employing the novel safety cushion design was validated using simulation software (CATIA). Sensitivity analysis was also used to confirm the design’s robustness. Results show that the manual blowing system using an accordion blower was the optimal design concept based on the selected evaluation criteria. In fact, the proposed design provides an acceptable RULA index value for the examined sitting postures and performed very safely in the biomechanics single action analysis.

## 1. Introduction and Literature Review

Humans spend lengthy durations sitting, causing their mental and physical health to be drastically affected. Several studies have investigated health issues related to prolonged sitting. Ref. [1] investigated the side effects of prolonged sitting and workers’ sedentary behaviors on physical health problems such as musculoskeletal disorders, oxygen deficit, inflammation, and many other problems that have a strong relationship with productivity. Recently, prolonged sitting was considered as a major causal factor for type 2 diabetes. Ref. [2] looked at the effect of reducing the stagnation of prolonged sitting on vascular functions, and the results indicated that more frequent and shorter breaks perform better than long and less frequent breaks for the vascular functions, which helps to mitigate the side effect of type 2 diabetes. The results of the study in ref. [3], which used 20 volunteers who were office computer workers as subjects, show that there may be a connection between prolonged sitting and musculoskeletal disorders and mental health issues. The study also examined the effect of prolonged sitting on the physical and mental health of computer workers. It was recommended that the stagnation of prolonged sitting should be avoided to eliminate such risks. As a direct cause of many health problems and a main outcome of prolonged sitting, obesity was cited by ref. [4]. The average sitting time of the sample involved in ref. [4] was found to be more than 3 h/day, and the outcomes of this study indicated that there is a strong positive relation between sitting time and being overweight. On the other hand, prolonged sitting can be considered as an indirect cause of mortality. Ref. [5] investigated cardiovascular health issues that are caused by prolonged sitting; 20 persons were examined, and the results showed that bouts of sitting for long durations without any breaks (standing, walking, engaging in exercise, etc.) lead to cardiovascular diseases and increased mortality risk. Although sitting persons periodically changed their sitting positions, the musculoskeletal disorders caused by lengthy sitting durations persisted [6]. 

Ergonomic considerations in chair design were the focus of several studies in the literature. As an example, Mistarihi, M.Z. et al. [7] developed a wheelchair that eliminates the awkward postures of disabled people and their assistants. In addition, the design reduces the work exerted by the assistants when transferring the disabled person from one location to another. Davis et al. [8] highlighted the disadvantages of utilizing home goods for office work by conducting a quality improvement evaluation, which found that such fixtures are not suited for long working hours and lead to awkward working postures and discomfort for the workers. In comparison to traditional chair designs, ergonomic chair designs offer practical solutions for reducing the harmful effects on chair users caused by prolonged sitting. Triglav, J. et al. [9] compared standard and multi-axis chairs (core chairs). They found that the latter delivers a considerable improvement in the physiology and cognition of the chair users. Ansari et al. [10] used anthropometric data on students during the educational process to develop an ergonomic chair design. The anthropometric data of surveyed students were as follows: for the seat, height: 44 cm, depth: 42 cm, and width: 42.15 cm; for the desk, height: 19–29 cm adjustable, depth: 51 cm, and length: 51 cm. The students’ musculoskeletal abnormalities were minimized by this proposed design. From a different point of view, simulation technology was utilized for a physical analysis of sitting persons with a finite element model (FEM) to determine the pressure distribution between the human and seat to design an optimal vehicle seat [11]. Ali, A.Y. [12] used different tools to design and develop a multipurpose shoeshine chair. This study aimed to determine customer needs, quality function deployment (QFD) methodology, concept generation process, concept selection, and, finally, cost analysis. The proposed model was competitive and succeeded in reducing the production cost of shoeshine chairs. Ref. [13] innovated a seat-integrated mobilization system that aims to reduce the muscle stiffness and discomfort of truck drivers with a dynamic mobilization system consisting of different air cushions with different functions in each that are aimed at providing a dynamic sitting position and preventing any negative impact from excessive sitting. Results showed that the motion activity of drivers who used this invention was increased, and their muscle stiffness as well as discomfort decreased significantly. Ref. [14] offered a cushion with a set of printed flex sensors to help with the user’s sitting posture and detect any unnatural or potentially harmful postures. If the user does not respond, the cushion will automatically correct the user’s posture using a microcontroller that is embedded inside the cushion. In a different application domain, a dental clinic, the patient must be in a symmetrical and straight position when reclined in the dental chair, while the dentist sits on a chair in an awkward posture. Ref. [15] provided a new design for a dental chair that aims to reduce the risk of MSDs to the dentist by incorporating two separated pillows that are able to be inclined and adjusted, reducing the pressure on the spine, eliminating back pain, and stimulating the blood circulation. 

Ref. [16] applied a concurrent engineering approach to modify the conventional wheelchair design with the aim of helping disabled and elderly people. The modified wheelchair design is equipped with an adjustable lever and bottle holder. To satisfy customer requirements, three alternatives were proposed: the multi-criteria decision-making (MCDM) approaches represented by the fuzzy analytical hierarchy process (AHP) and quality function deployment (QFD), and value engineering to optimize the selection process and maintain the quality of the selected design as well as possible. 

Several types of research investigated the effect of unhealthy sitting situations to propose different solutions aiming at reducing the negative impact on the physical health of the occupants subjected to lengthy sitting durations [17,18]. Yasuhiro Otoda et al. [19] provided a modified chair to measure the sitting situation of the workers, classify their sitting situation in different categories, and notify the sitting person when to change their posture. However, Daneshmandi H. et al. [20] surveyed 447 office workers to investigate the effects of prolonged sitting. Findings indicated that there was an increase in fatigue. Additionally, the musculoskeletal disorders (MSDs), signs of high blood pressure, and awkward body postures in the knees, shoulders, thighs, and lower back all significantly deteriorated in the workers. Different strategies were put forth to counteract the negative effects of prolonged sitting times. Teng Teng [21] proposed a device (chair design) to help people maintain a healthy sitting situation and keep them safer and more comfortable. Table 1 summarizes the results of the related research.

Several researchers utilized MCDM approaches in various applications including design. Dang et al. [22] integrated the fuzzy analytical hierarchy process (FAHP) and the fuzzy technique for order preference by similarity to the ideal solution (FTOPSIS) to evaluate and sort the susceptibility data. The findings of this study showed that education, healthcare, quality of life, and social democracy were the most important markers to measure progress. Furthermore, regarding environmental sustainability, the parameters of water quality and garbage disposal were placed first and second, respectively. Mistarihi, M.Z., et al. [9] combined QFD and the fuzzy analytical network process (FANP) to determine the most critical variables in the suggested wheelchair design. Results showed that material quality was selected as the most influencing engineering variable. The evaluation of the wind turbine for the selection was conducted by ref. [23] using hesitant fuzzy AHP-TOPSIS and a variety of qualitative and quantitative parameters. Based on the results of this investigation, the A3 wind turbine type was selected as the best solution. Keshteli and Davoodvandi [24] involved FAHP and FTOPSIS in the QFD approach to eliminate the vagueness and uncertainty of the traditional QFD approaches. In this study, the QFD tool was used to involve customer feedback in the design process to maximize “customer happiness”. FAHP was used to weigh customer requirements, and FTOPSIS was used to prioritize the design requirements of the HoQ component. A ceramic tile plant was taken as a case study, and an essential design requirement was the thickness and dimension described by D4, while the design was considered the most critical issue among the clients’ requirements. Yucesan and Gul [25] used the FAHP and FTOPSIS Pythagorean to assess hospital service quality. The Pythagorean FAHP was used to weigh 32 criteria, while the Pythagorean FTOPSIS was used to rank the hospitals. Thirty-two service quality criteria for two public and one private hospital were investigated. The most crucial evaluation criterion was the professional skill of the medical personnel, and hospital number one was chosen as the highest service quality provider. Ding, Y., et al. [26] utilized the Fuzzy AHP to override the vagueness of the experts and make the right decisions to solve the transportation corridor problems. The integration between the decision-making approaches is helpful in determining the critical quality characteristics of the new products and prototypes. Liu [27] operated the integration between the fuzzy QFD and fuzzy MCDM to help the designers and developers to optimize the quality of products and determine the product weakness, robustness, market position, and sales point of their products.

The integration between the DM tools provided several solutions for the product producers. Milunovic Koprivica and Filipovic [28] performed the fuzzy QFD to maximize the quality characteristics of the boilers and keep their products competitive in the market. On the other hand, Olabanji., et al. [29] performed the integration between the FAHP and fuzzy weighted average to select an optimum design of the reconfigurable assembly fixture RAF from different design concepts. On the other hand, Keramati, A. et al. [30] integrated the QFD with the ANP to make a cost analysis to optimally select the supplier in the automotive industry based on the importance degree of the CNs and ECs then. A tradeoff between the subjective factor measurements and the objective factor measurements was involved in supporting the decision.

Previous research proposed solutions and designs for prolonged sitting physical and mental health problems in specific domain applications. Earlier research used different groups, including, disabled and older people, students, drivers, dental patients, shoe polishers, babies, and office workers, as focus groups for their research and to create solutions to make their seating conditions safer and more comfortable. However, there was a lack of ergonomic solutions to the skin problems due to prolonged sitting. Not so many unique designs are proposed for lengthy sitting to reduce its negative impact on the skin and the muscle of the users. The presented research provides “a chair attachment cushion with optimal air blowing technique” that includes air cells embedded inside the cushion used to instantaneously reduce the contact area between the back of the chair and the back of the occupant. The air cell is filled by air with different operating methods, leading the occupant to gently move forward, apart from the back of the chair, allowing the surrounding air to enter the space between the back of the chair and the back of the occupant, leading the sweats to be eliminated so that the problems caused by sweat reduced accordingly. Additionally, by making it simpler for decision makers to evaluate connected elements in an unpredictable environment, this study expands the use of the FAHP approach, which is not frequently used in the product development/selection industry. It is a method of multicriteria analysis based on additive weighting, where several relevant attributes are represented by their relative relevance. FAHP is employed in this research work due to its theoretical comprehension, usability, and robustness of outcomes that have been proved in practice [7,22,23,24,25,26,27,28,29,30,31]. This work is organized into the following sections: a brief explanation of multi-criteria decision making approach and the proposed design is included in Section 2. The selection of evaluation criteria, data collection, and ergonomics analysis are introduced and discussed in Section 3. Section 4 presents the findings and future research.

## 2. Materials and Methods

### 2.1. Study Design and Data Collection

The data of the presented research has been divided into two categories: the first one was related to the customers (users of the proposed design) where they were surveyed online using the “Google Forms” platform. The collected data involved the investigation of the impact of the prolonged sitting duration on the health of participants. Additionally, the potential customers were surveyed to understand their need to make the proposed design of the seating attachment cushion compatible with their requirements. The second category of data was collected from the expert team who fill out the evaluation matrices based on their opinions and experiences to select the optimal alternative of the proposed design based on the results of the selected MCDM approaches.

#### 2.1.1. Study Participants

Four hundred four participants across the service, students’ universities, and manufacturing industries in Jordan were recruited to participate in this study. They have been hypothesized to investigate the impact of prolonged sitting on their health, especially their skin. The null hypothesis denoted by ‘H_0_′ refers to (there is no negative impact on the participant’s health due to the lengthy sitting duration on the chair). The alternative hypothesis describes the inverse case of the null hypothesis, denoted by ‘H_1_′ to refer to (there is a negative impact on the participants health due to the lengthy sitting durations). Due to the nature of the examined data and the main goal of the statistical analysis, a binary logistic regression analysis was utilized to check the hypothesis of the impact of the lengthy sitting duration on the participants’ health. In this statistical test, a response variable or dependent variable (skin sore or pressure ulcer) is related to two independent variables (sitting on the chair for less or more than four hours). A summary of the respondents’ survey response characteristics is presented in Table 2.

#### 2.1.2. The Expert Team

Due to the nature of the proposed designs, the selection of the expert team (decision makers) should take the scientific background, the experience, and the ease of accessibility to each expert into consideration, which facilitates and optimizes the filling of the evaluation matrices and related questionnaires. In the presented study, an expert team from three local universities is targeted to be the evaluators of the proposed designs. The team consists of the academic staff of the industrial, biomedical, and mechanical engineering departments of the Jordan University of Science and Technology, Yarmouk University, and Al Huson College University, in addition to the engineering workshop technicians. The overall academic staff size for such universities was about 120, categorized into different specialties (doctorate in mechanical, industrial, biomedical engineering, mechanical technicians, mechanical and industrial engineering, physiotherapist, and fabricators). Each of these experts evaluates the four design concepts from their specialty point of view on the proposed evaluation criteria. The characteristics of the experts that evaluate the criteria and the proposed designs are summarized in Table 3.

### 2.2. Multi-Criteria Decision-Making Approach

The multi-criteria decision-making approaches have been utilized in a wide variety of applications. These approaches can simultaneously involve several evaluation criteria to make decisions by considering the decision-makers’ opinions. Making the right decision in MCDM is different from the mathematical calculations since the optimal solution is extracted based on several considerations and other decision-makers’ opinions [32]. 

Many decision-making problems have a vague and uncertain environment that significantly affect the decision-making process, since the decision makers have different experiences, emotions, knowledge, and priority management. Fuzzy theory is involved in the process to deal with the uncertainty and vagueness of the process of the DM [33].

#### 2.2.1. Fuzzy Analytical Hierarchy Process (FAHP)

The most frequently used approach of the MCDM is the AHP due to its simplicity and ability to deal with most DM problems. The core of the AHP process is the pairwise comparison. The decision is obtained by weighing the alternatives based on several evaluation criteria concerning expert opinions. The regular AHP is unable to deal with the inaccuracy and subjectivity of pairwise comparison, so the FAHP is introduced. The fuzzy number is used instead of a crisp value in the fuzzy approach to deal with the imprecision and uncertainty of the decision makers. These fuzzy numbers describe the behavior of the examined alternatives based on the selected criteria. In this paper, the fuzzy trapezoidal number is used, which is expressed as:

(1) $$\tilde{m}=\left(a,b,C,D\right)$$

The membership function of the fuzzy trapezoidal number can be expressed as:

(2) $$\mu_{\tilde{m}}\left(x\right)=\left\{\begin{array}{c} \frac{x-a}{b-a}\;a\leq x\leq b\\ 1\;b\leq x\leq c\\ \frac{d-x}{d-c}\;c\leq x\leq d\\ 0\;otherwise \end{array}\right.$$

**Figure 1:**
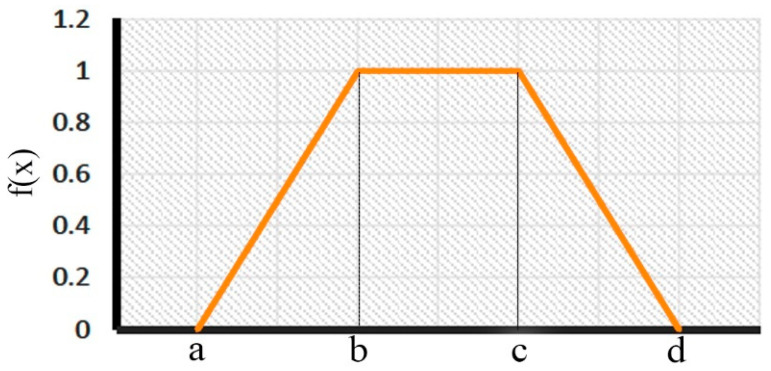
The trapezoidal fuzzy membership function.

Moreover, the graphical plot of the membership function of the trapezoidal fuzzy number is shown in Figure 1.

#### 2.2.2. Defuzzification

It is challenging to manage the trapezoidal fuzzy numbers, and there is some vagueness with the approximation of the worth of those numbers. To override this problem, a mathematical operation (defuzzification) is used to convert these numbers into a crisp value. Where the crisp value enables the reader to imagine the weight of such a number easily, Equation (3) is used to make the conversion between the fuzzy values into a crisp value.

(3) $$\begin{array}{l} N=\frac{b+c}{2}+\left[\left(d-c\right)-\left(b-a\right)\right]\\ \;=\frac{\left(a+2b+2c+d\right)}{6} \end{array}$$

### 2.3. Calculating the Weights of Criteria

#### 2.3.1. Fuzzy AHP

The first step in this approach is consistency checking to ensure that the ranking process goes in the right direction. The fuzzy comparison matrices are considered consistent if the related crisp comparison matrix is consistent. The consistency checking of the pairwise comparison matrices has been done by applying the following steps:

1. Creating the crisp pairwise comparison matrix.

(4) $$\tilde{x}=\left[\begin{array}{ccc} {\tilde{x}}_{11} & {\tilde{x}}_{12}\ldots & {\tilde{x}}_{1n}\\ {\tilde{x}}_{21} & {\tilde{x}}_{22} & {\tilde{x}}_{2n}\\ \tilde{x}n_{1} & \tilde{x}n_{2} & {\tilde{x}}_{nn} \end{array}\right]$$

where $\tilde{X}$_ij_ is the scale of *T_i_* compared with *T_j_*, ($\tilde{X}$_ij_)^−1^ is the scale of *T_j_* compared with *T_i_* (inversion), where *T_i_* represents the value in the ith row, *T_j_* represents the value in the jth column.

(5) $${\tilde{X}}_{\mathrm{ij}}=(l_{ij},m_{ij},n_{ij},s_{ij})$$

(6) $${\tilde{X}}_{\mathrm{ij}}=({\tilde{X}}_{\mathrm{ij}}{)}^{-1}=(s_{ij-1},n_{ij-1},m_{ij-1},l_{ij-1})$$

2. Calculating the consistency ratio using the following formulas.

*CI* = (λ_*max*_ − *n*)/(*n* − 1) (7)

*CR = CI/RI* (8)

where *λ_max_* represents the largest eigenvalue of the matrix; (*n*) is the matrix size and RI represents the random index shown in Table 4.

The matrix is considered a consistent matrix if the *CR* ≤ 0.1. Otherwise, the matrix will be rejected.

3. Calculating the weights of criteria by finding out *αj*, *βj*, *γj*, *δj*, *α*, *β*, *γ*, and *δ* which are calculated using Equations (9) and (10):

(9) $$\alpha_{j}={\left[\Pi_{j=1}^{n}l_{ij}\right]}^{1/n},\;\beta_{j}={\left[\Pi_{j=1}^{n}m_{ij}\right]}^{1/n},\;\gamma_{j}={\left[\Pi_{j=1}^{n}n_{ij}\right]}^{1/n},\;\delta_{j}={\left[\Pi_{j=1}^{n}s_{ij}\right]}^{1/n}$$

(10) $$=\sum_{j=1}^{n}\alpha_{j},\;\beta =\sum_{j=1}^{n}\beta_{j},\;\gamma =\sum_{j=1}^{n}\gamma_{j},\;\delta =\sum_{j=1}^{n}\delta_{j}$$

Then, the geometric mean value $\tilde{r_{j}}=(\alpha_{j},\beta_{j},\gamma_{j},\delta_{j})$, and the inverse of the $\tilde{r_{j}}$ can be computed using Equation (11).

(11) $${\tilde{r_{j}}}^{-1}=(\delta^{-1},\gamma^{-1},\beta^{-1},\alpha^{-1}).$$

After that, the weight of each criterion is calculated using the following Equation:

(12) $${\tilde{w}}_{j}=\left(\alpha_{j}\delta^{-1},\beta_{j}\gamma^{-1},\gamma_{j}\beta^{-1},\delta_{j}\alpha^{-1}\right);j\in \left\{1,2,\ldots ,n\right\}.$$

Finally, the fuzzy weight vector $\tilde{W}$ is shown as a single row matrix:

$$\tilde{W}=\left[\tilde{W1}\tilde{W2}\ldots .\tilde{Wn}\right]$$

#### 2.3.2. Fuzzy TOPSIS

One of the most popular MCDM strategies is the FTOPSIS method. This strategy is focused on choosing the best option that, in accordance with the evaluation criteria, is the furthest away from the worst solution and the closest to the best answer. The TOPSIS method aims to find the weights of each criterion by calculating the average of the evaluation of the decision makers and applying the following procedure. 

Let (*k*) be several decision makers, each of which evaluates each design. Then, their evaluation represents trapezoidal fuzzy number E; E = *(a_k_, b_k_, c_k_, d_k_)*, and the average E_avg_ = (a_avg_, b_avg_, c_avg_, d_avg_), where:

(13) $$A_{\mathrm{avg}}=\min\;\{ak\},\;B_{\mathrm{avg}}=1/k\;\sum_{k=1}^{k}b_{k},\;C_{\mathrm{avg}}=1/k\;\sum_{k=1}^{k}c_{k},\;D_{\mathrm{avg}}=\max\;\{d_{k}\},$$

### 2.4. Ranking the Designs

In this step, the ranking of the proposed designs is the same as established by the fuzzy evaluation matrix. The evaluation value ${\tilde{f}}_{j}$ of the attribute $f_{j}$ (the evaluation of the decision group for the design performance) can be obtained by:

(14) $${\tilde{f}}_{j}=\frac{1}{k}({\tilde{f}}_{j}1+{\tilde{f}}_{j}2+\ldots +{\tilde{f}}_{j}k)$$

where ${\tilde{f}}_{j}$ = (l, m, n, s) is a fuzzy number denoting the evaluating value of the attribute (*f_j_)*, given by decision maker Dk, and 1 ≤ j ≤ n. Additionally, the fuzzy evaluating matrix can be obtained as

$$\tilde{f}=[{\tilde{f}}_{1}{\tilde{f}}_{2}...{\tilde{f}}_{n}]$$

The next step is to find out the fuzzy evaluating vector, based on the fuzzy evaluating matrix and the criteria weights. The fuzzy evaluating vector for each design alternative can be obtained according to the following formula:

(15) $$\tilde{Z}=[\left({\tilde{w}}_{1}\times {\tilde{f}}_{1}\right)+\left({\tilde{w}}_{2}\times {\tilde{f}}_{2}\right)+\left({\tilde{w}}_{3}\times {\tilde{f}}_{3}\right)+\ldots +\left({\tilde{w}}_{j}\times {\tilde{f}}_{j}\right)/({\tilde{w}}_{1}+{\tilde{w}}_{2}+{\tilde{w}}_{3}+\ldots +{\tilde{w}}_{j})]$$

### 2.5. The Proposed Designs

In this subsection, the analysis of the customer surveys fructifies the novel design of the chair attachment cushion with four alternatives to the chair user contact area reduction system. The way that these systems operate is different. Nonetheless, they all lead to the same outcome; minimizing the area of contact between the chair and the user to prevent the development of skin health issues such skin sores, pressure ulcers, and numbness. These systems of instantaneous contact area reduction are the manual blowing system using the accordion blower, the manual blowing system using the pedaling mechanism, the automatic blowing system using the gas expanding principle, and the automatic blowing system using a driving motor. The cushion is the same for all four systems, but with different operating methods to blow the air cell inside the attachment cushion. In the following subsections, each system will be introduced with descriptive illustrations and (CAD) drawings. 

#### 2.5.1. The Manual Blowing System Using the Accordion Blower

**Figure 2:**
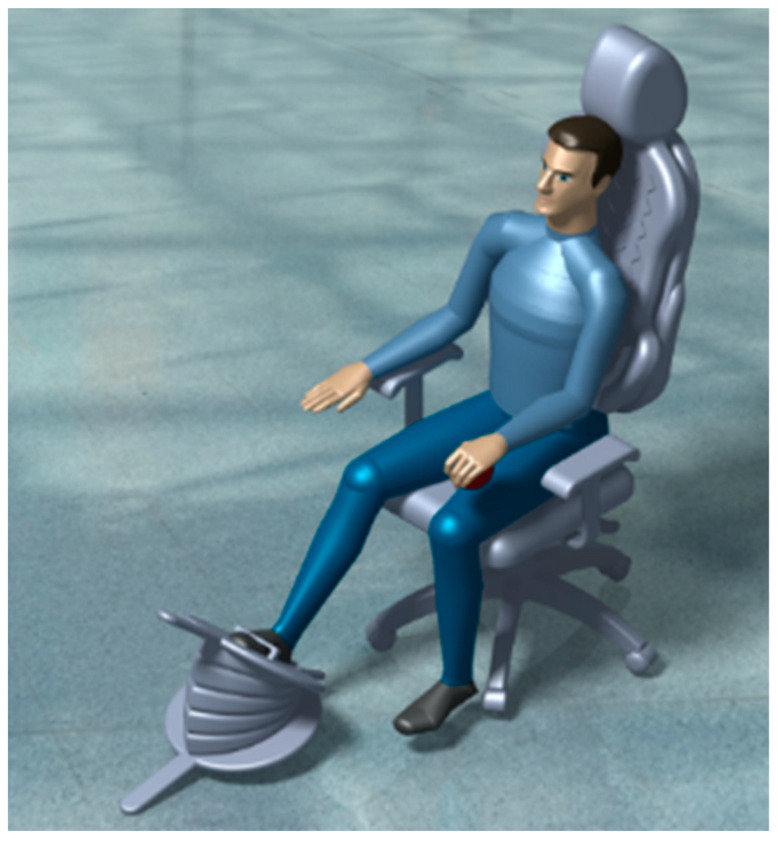
The seated person is using the accordion blower system.

In this system, the user blows the air pillows manually by pressing the accordion blower down with their feet. Through this procedure, compressed air will be pumped to the air pillows so they can be blown. The user needs to push the blower several times to fill the air pillows to reduce the contact area between the user and the chair, as demonstrated in Figure 2.

**Figure 3:**
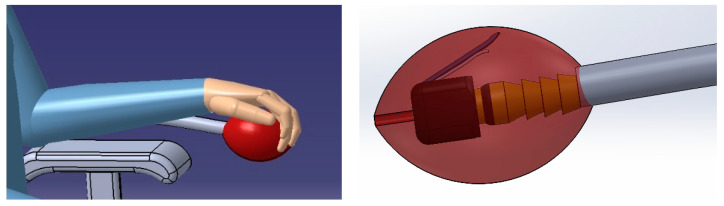
Venting part in hand, including the manual air pump.

The compressed air is pumped to the air pillows through an air hose using a one-way valve which shuts the air inside the pillows. However, if the user wants to vent such pillows, they should compress a comfort ball by hand, allowing the prisoned air to escape from the pillows. This ball contains an air nozzle to apply such a function, as shown in Figure 3.

**Figure 4:**
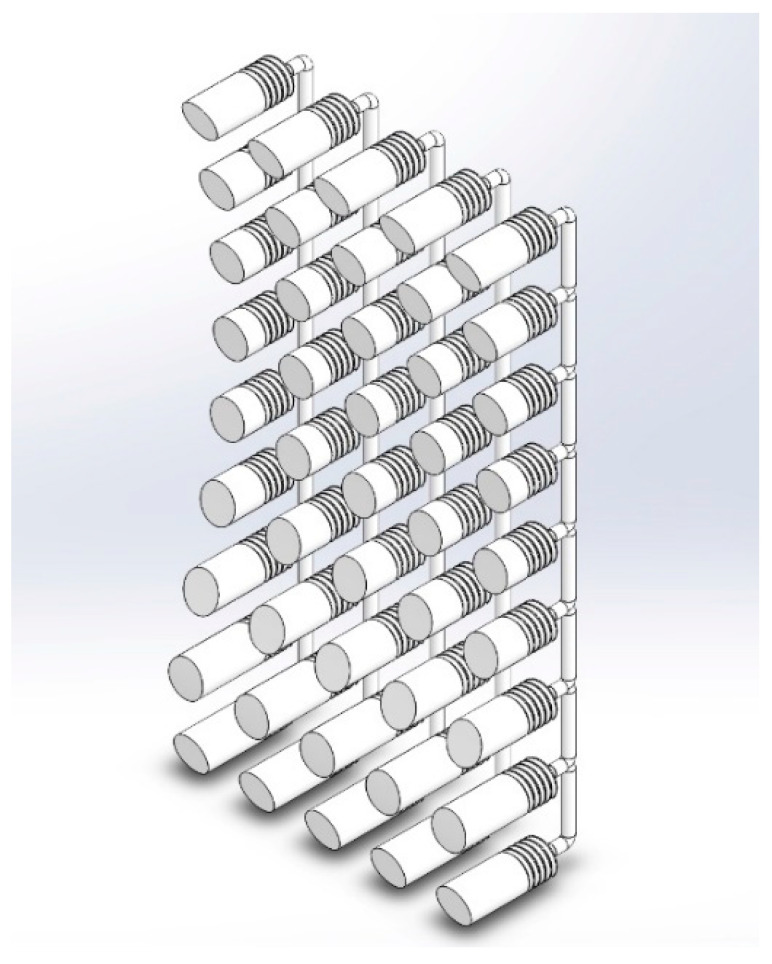
The design of the air cell system mimics the human spine.

The air cell system is designed based on ref. [34] to push the back of the occupant to a position that satisfies the primary goal of the research, which is based on instantaneous contact area reduction. The reduction will be performed instantaneously to prevent any adverse effect on the pressure distribution (see Figure 4).

**Figure 5:**
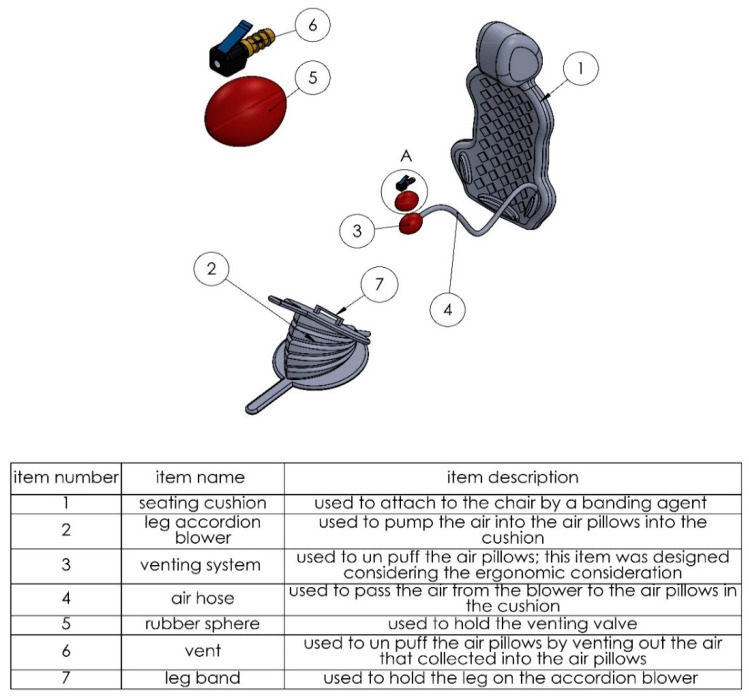
The parts of the manual blowing system using the accordion blower.

When the user needs to vent the air cell and refers the cushion to the normal situation, they use the comfort ball to compress the venting part attached to the cushion to allow the imprisoned air to escape from such cell. The venting part contains an air nozzle inside it, designed for such a purpose. The parts of the manual blowing using an accordion blower are shown in Figure 5. 

#### 2.5.2. The Manual Blowing System Using the Pedaling Mechanism

**Figure 6:**
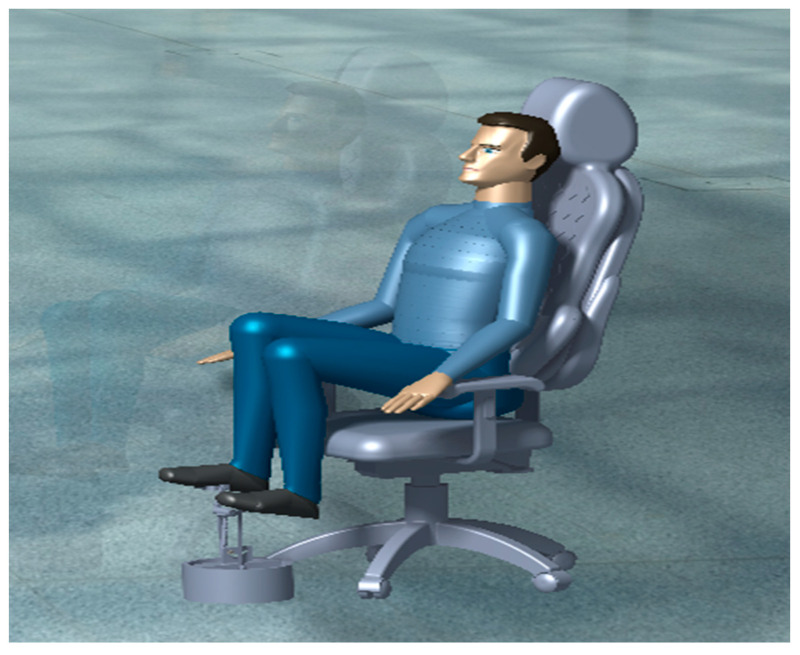
The manual blowing system with the pedaling mechanism.

**Figure 7:**
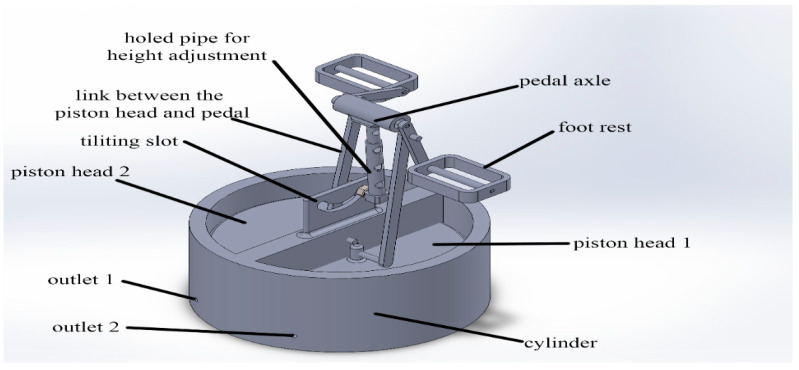
The parts of the pedaling mechanism.

As seen in Figure 6, the user blows the air cell manually in this operating system by exercising (pedaling) to pump air into the air cell. The design of the pedaling system contains different parts, as demonstrated in Figure 7. The design consists of two separated champers, each of which is connected to a set of air pillows inside the air cell by a hose, two piston heads are used to compress the air inside the chambers and pump it to the air cell. Two linking bars join the piston heads to the pedal cranks. Modified pedal cranks are used to translate the rotational movement of the pedals to a translational motion of the piston heads to achieve the blowing operation, and two foot rest pedals are used to locate the user’s feet. 

#### 2.5.3. The Automatic Blowing System Using the Gas Expanding Principle

**Figure 8:**
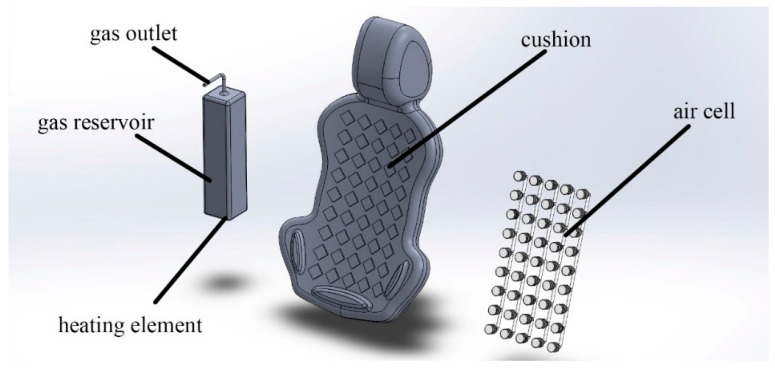
The automatic blowing system uses the gas expanding principle.

In this system, expanded air that is pumped from the air container through an air hose that has been passed through a valve blows the air cell. When air is heated, an air container connected to a heating source forces the air molecules to expand, forcing them to escape the container and enter the air cell. The gas container is designed to be fully insulated from heat and electricity to prevent direct contact with the user. The heating element is connected to an automatic shutdown switch to reduce energy consumption, and it is also connected to a limit switch to prevent overload. The parts of the automated system using the gas expanding principle are shown in Figure 8. 

#### 2.5.4. The Automatic Blowing System Using a Driving Motor

**Figure 9:**
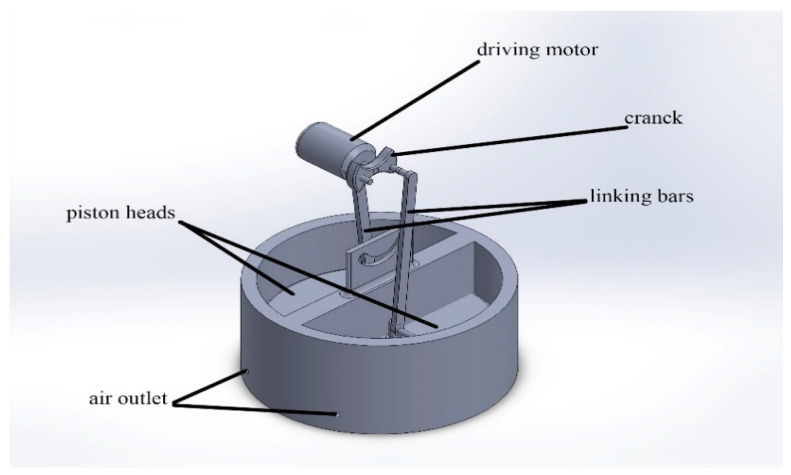
The parts of the automatic puffing system using a driving motor.

In such a system, the air cell is blown by the same method as the pedaling system; however, the piston heads are driven automatically by using a driving motor connected to such heads. The motor drives the piston heads, which are used to pump the air inside the air cell through an air hose. The parts of such a system are shown in Figure 9.

An expert panel assessed these four designs using the FTOPSIS and FAHP techniques to choose the best option based on a number of assessment criteria; these criteria will be discussed in Section 3.1.

### 2.6. Product Design Consideration

#### 2.6.1. Design for Ergonomics

The proposed designs consider the users’ anthropometric measurements, such as their weight and length. They fit a large segment of the users and make it compatible with different types of chairs. The manual system imposes the users to do some activities to perform the product. These activities require the user to manually blow and vent the cushion’s air pillows, which stimulate the user’s blood circulation, break the stagnation of the lengthy sitting situation, and achieve the aim of instantaneous contact area reduction.

Other ergonomic features of the proposed designs related to the geometry of such a design concept as the shape of the venting part, which was an elliptical shape, and the shape of the air pillows after the blowing process designed to follow the shape of the human spine, the type of material of the cushion to fit the user’s skins, the procedures of the blowing, and venting the air pillows of the manual system. These features and the overall design concept should fit the user’s anthropometric data.

**Figure 10:**
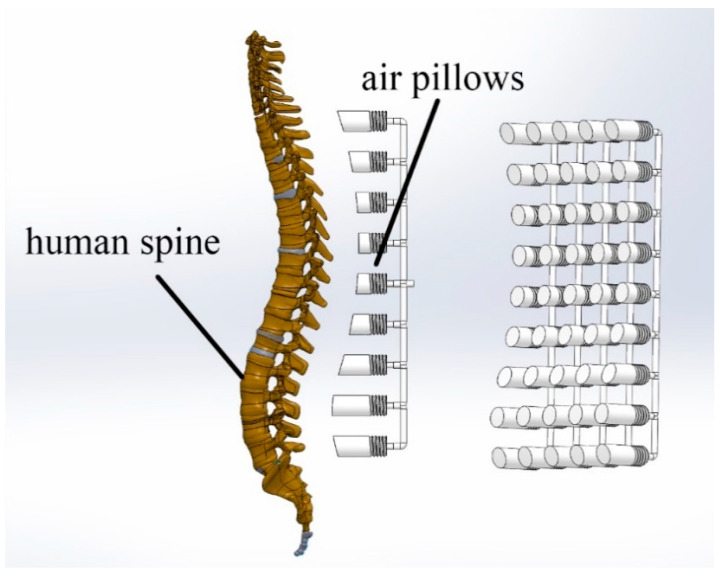
The air cell design is based on the human spine curve.

The air cell was designed for each air-blowing technique to follow the shape of the human spine to conduct an optimal pressure distribution on the human back, paying attention to the main aim of the research, which was instantaneously reducing the contact area between the chair and the user. Figure 10 shows how the air cell embedded inside the cushion follows the human spines. In the following subsections, the ergonomic considerations of the manual systems are introduced since the automatic ones do not require any effort from the users to perform the cushion system.

#### 2.6.2. Design for Safety

The proposed designs consider the safety issue as their primary consideration. Safety failure will be introduced, especially for the automatic system using the gas expanding principle designed to heat the air to be expanded and filled the pillows. It will be heated to about 60 ± 5 °C. The heating element will be insulated well to facilitate dealing with it and keep users from any expected hazards. The operation scenario does not always go right. It will help if you take the worst-case scenario with a significant possibility of keeping the design safe for both the users and the functions of the design. In the proposed designs, the failure of the automatic system, for example, the over-heating of the gas, electrical short circuit, over-load case, and gas leakage. For the gas overheating, a pressure relief valve will be attached to the system to vent the excessive amount of the gas when it exceeds the required volume where the gas is non-toxic (air). To protect from electrical shock, the electrical cables should be insulated well from its surroundings, a set of plug adaptors should achieve the electrical connections, and the electrical circuit should have electrical fuses to prevent any shock. To avoid the overload condition, an automatic shut-off switch should be added to the electrical circuit to keep the heating system at the desired level and to keep the energy saved from overconsumption. Finally, for the gas leakage, the selected gas should be safe for the user in the case of leakage (non-toxic gas, not harmful when the user inhales some of it). The gas chosen for the proposed design was air.

#### 2.6.3. Design to Fit the Human’s Anthropometric Measurements

In this aspect, most of the surveyed sample will be focused on translating their anthropometric measurements to the chair cushion. The most critical measures in the proposed design are the customer’s weight and length. The vast majority of the 404 surveyed persons have an average length of 150–170 cm and an average weight of about 60–85 kg. The height of the cushion was 55 cm, which is acceptable for both the chair and the user. On the other hand, the thickness of the upholstery material is set to withstand a weight of about 95 kg, so the thickness of the cushion was set to be 7 cm. 

### 2.7. The RULA Analysis

The proposed design of the chair attachment cushion should be analyzed ergonomically to check its compatibility with the user. The ergonomic analysis in the presented research will be performed by applying the rapid upper limb assessment (RULA) approach to assess the sitting postures of the user on the selected design. Using the resulting RULA score, it can be decided whether the design is suitable for the user and satisfies the health and safety considerations. The RULA analysis is a useful tool for estimating the MSDs that humans experience when engaging in activities such as everyday chores, sports, sitting positions, and other situations [35]. This tool gives a score out of 7 (the highest score of risk), so a procedure should be taken to eliminate that score to be acceptable and reduce the risk factor of such posture. The (RULA) analysis, as well as the biomechanics single action analysis of the selected design, will be performed, explained, and discussed in the results section of this study.

## 3. Results and Discussion

### 3.1. The Selection of Evaluation Criteria

Several criteria are involved in the evaluation process to enhance the selection of the optimal alternative to the examined designs and make the evaluation process more accurate and inclusive. In this section, the evaluation criteria of the chair attachment design will be introduced; these criteria will be summarized from the previous related literature and experts’ opinions.

1. After analyzing the prior literature [7,36,37,38,39,40,41,42,43,44] to extract the evaluation criteria of the proposed design and making some modifications to them, the most valuable criteria that are consistent with the studied designs will be introduced as follows. Performance: it introduces the time of puffing the air cell. That is the speed of the puffing operation;
2. Design: the strengths and weaknesses of the examined designs and the ability to produce such designs;
3. Expected lifetime: how long the product serves without any problem or any initiation of failures;
4. Comfortable feeling: how much the customer feels comfortable when using the product;
5. Complexity: the method of production used to obtain the examined designs. If the design is shortened to the traditional manufacturing process, it will not be classified as complex. However, suppose the design requires any of the advanced manufacturing technology to be produced, such as the non-traditional or additive manufacturing system. In that case, it will be classified as a complex design;
6. Cost: it includes the cost of material, production, and equipment;
7. Assembly and disassembly: the ease of assembling the final product from its components;
8. Safety: how the customer feels safe when using the product.

### 3.2. Data Analysis

Forty-nine professionals in the fields of designing, manufacturing, and engineering evaluated the four alternative designs according to the eight chosen criteria to determine which was best.

#### 3.2.1. Fuzzy AHP

Each of the 49 decision makers has been asked to fill out the evaluation questionnaire. The comparison evaluation matrices were performed according to the linguistic variables and their corresponding fuzzy numbers and after making sure that each matrix was consistent. The overall average matrix of the 49 matrices is presented in Table 5. 

#### 3.2.2. Fuzzy TOPSIS

In the FTOPSIS method, the survey was distributed to the decision makers (experts) to evaluate the selection process criteria based on their preferences according to a scale used for such purposes, and the results were shown in Appendix A: Table A1.

#### 3.2.3. Design Concepts Evaluation

In this step, the four proposed design concepts were subjected to an evaluation process based on forty-nine experts’ opinions and preferences to select the optimal design. This evaluation process was done according to the concept performance concerning the evaluation criteria point of view. The evaluation process results for each design concept were introduced in Table A2.

### 3.3. Calculations

In this subsection, the calculation processes will be introduced (fuzzy AHP, fuzzy TOPSIS, fuzzy evaluating process, and fuzzy evaluating vector).

#### 3.3.1. Fuzzy AHP

Consistency checking is an essential operation to check the feasibility and the logistics of the comparison matrix. Equations (7) and (8) and Table 4 are utilized to check the consistency ratio for the average comparison matrix.

- The maximum eigenvalue in the matrix (λ_max_) was calculated using MS Excel software, and found to be 8.40547033;
- The random index was found to be 1.41 since the matrix size was 8;
- Applying Equation (7) to get CI (consistency index) which was found to be 0.0579. Using Equation (8), the consistency ratio CR was found to be 0.0411, less than 0.1. So, the CR is acceptable, and the average criteria evaluation matrix is consistent;
- Converting the crisp matrix into a fuzzy matrix: since the fuzzy values are needed to complete the calculations, the crisp values were converted into fuzzy values based on the scale of the linguistic expressions and related scale of the trapezoidal fuzzy numbers, and the resulting matrix is shown in Table 6;

- Calculating the weights of the criteria: based on the pairwise comparison matrix and using Equation (9); the coefficients of the criteria weight are shown in Table 7:

As a result, the summations of α are β, γ, δ, and the inverse of the summation of the coefficients of the criteria weights are presented in Table 8 and Table 9, respectively.

Next, the weights of the criteria (Equation (12)) and the defuzzification (Equation (3)) are shown in Table 10. 

After making the defuzzification of the trapezoidal weights, the rank of the evaluation criteria using the fuzzy AHP process will be as follows: safety, design, cost, performance, comfort feeling, expected lifetime, complexity, and assembly and disassembly. 

#### 3.3.2. Fuzzy TOPSIS

By converting the linguistic expressions found in Table A1 into fuzzy numbers according to the scale of the linguistic variables and their corresponding fuzzy numbers, the resulting criteria evaluation matrix are shown in Table A3.

Using Equation (13), the weights of the eight criteria utilizing the fuzzy TOPSIS method are shown in Table 11.

Next is applying Equation (3) to the trapezoidal numbers in Table 11. The rank of the criteria using the fuzzy TOPSIS method is as follows: safety, cost, comfort feel, performance, design, expected lifetime, assembly and disassembly, and complexity.

After constructing the fuzzy evaluating matrices for both approaches, the fuzzy evaluating vector is extracted, allowing one to rate the four options and select the best design concept out of the four suggested ones. Based on Equation (15), the fuzzy evaluating vector was calculated. Table 12 summarizes the fuzzy AHP results because the Z score indicates the weight of each design concept.

The exact process was repeated, but this time the criteria weights were based on the values found using the FTOPSIS approach, and the outcomes are displayed in Table 13. The manual blowing system with the accordion blower was found to be the optimum design concept after performing MCDM calculations with the fuzzy AHP (Table 12) and fuzzy TOPSIS (Table 13) techniques.

#### 3.3.3. Sensitivity Analysis

The sensitivity analysis term can be described as the study of the behavior of uncertainty in the inputs of a model, and how it behaves due to the changes in the values of such inputs. The sensitivity analysis’s main purpose is to demonstrate how the chosen decision changed when the values of the inputs were altered. In the presented case, the sensitivity analysis approach was conducted using MS Excel software to consider the variation of the evaluation criteria’ importance weights in evaluating the four proposed designs. Therefore, six combinations for the FAHP method and six combinations for the FTOPSIS method were performed using only the most four weighted criteria for the two approaches.

##### FAHP Sensitivity Analysis

The sensitivity analysis was performed by taking two combinations of criteria once at each experiment, and the results of the sensitivity analysis of fuzzy AHP outputs are summarized in Table 14.

##### FTOPSIS Sensitivity Analysis

The sensitivity analysis of fuzzy TOPSIS outputs is summarized in Table 15, where the same procedure was repeated as the fuzzy AHP process. 

The selected alternative design concept number four, “manual blowing system using the accordion blower”, maintains the same rank for all 12 experiments, as shown by the sensitivity analysis results for both methods (FAHP and FTOPSIS). This indicates that the chosen alternative is robust and insensitive to changes in the most weighted criteria.

### 3.4. Quality Function Deployment

Determining the engineering features’ highest weighted value extracted from the quality function deployment and its house of quality. The quality function deployment approach will be mapped to the proposed design to understand the basic customer requirements and translate them into engineering characteristics to determine the most weighted value of such requirements. The translation from the customer requirements into engineering characteristics of the selected design concept will be achieved by utilizing the house of the quality template. The primary customer requirements of the proposed design are price, safety, appearance and aesthetic features, ease of use, repair, and cleaning, and performance, as well as no conflict with the main functionality of the chair. These customer requirements are then translated to a set of engineering characteristics: total cost, design complexity, type of material, production complexity, and air cell puffing time. Two hundred forty-six customers were surveyed to fill out a questionnaire about their preferences, and the results are summarized in the form of the HoQ template as shown in Table 16. The legend of the HoQ is presented in Table 17.

The HoQ template results show that the most weighted value of the engineering characteristics was the “cost of equipment” with a ratio of (24.2%). So, the designers should take the cost of equipment as an important factor in the design process. 

**Figure 11:**
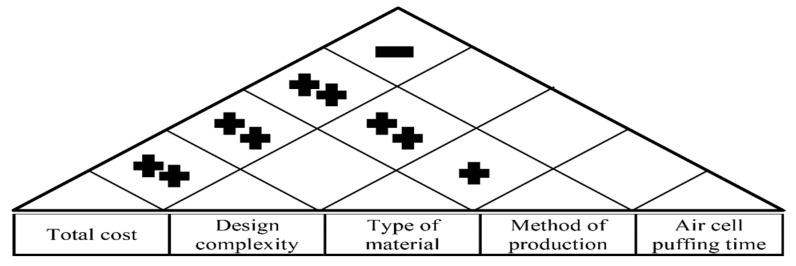
The relationship between engineering characteristics.

The connection between engineering attributes was presented in Figure 11.

**Figure 12:**
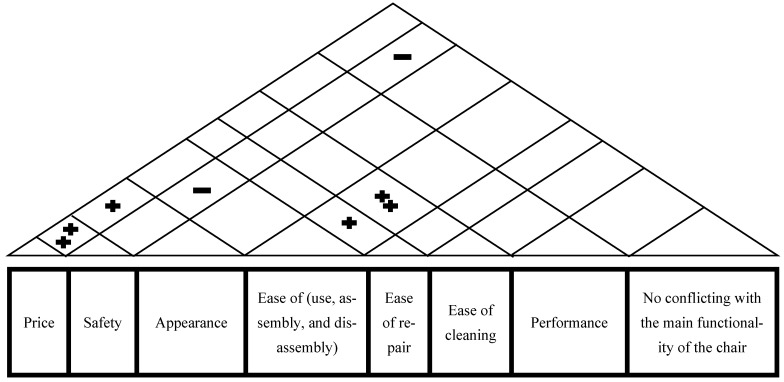
The customer requirements relationship matrix.

For the relationship between customer requirements, the matrix was presented in Figure 12.

As a result of the QFD, the designer engineers should concentrate on the “cost of equipment” to improve the quality of the suggested designs. This conclusion is plausible given that the quality of the suggested designs is mostly dependent on the quality of their constituent parts, which is directly correlated with their cost (the higher the quality, the more expensive the component).

### 3.5. Ergonomics and Biomechanics Analysis

Evaluating ergonomic risks during the design phase allows for the early identification of problems and the application of corrective measures, which is more efficient and less expensive than a later assessment of these risks. An ergonomic analysis to validate the safety and health considerations of the user is required. For making such an analysis in the early assessment of ergonomic conditions, the CATIA software will be utilized in a virtual environment of the product life cycle. Using simulation software reduces time and thus, financial requirements, in the phases of research, development, experimenting, and the manufacturing of a technical object. The sitting posture of the user while they are using the seating cushion and the accordion blower will be analyzed, and the results will be presented first as a RULA score, which indicates how the user’s safety and health considerations are satisfied. 

**Figure 13:**
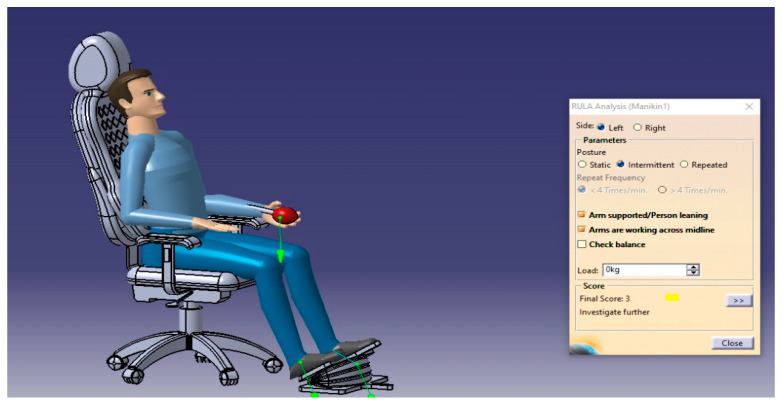
The RULA analysis output of the body’s left side in the intermittent posture.

**Figure 14:**
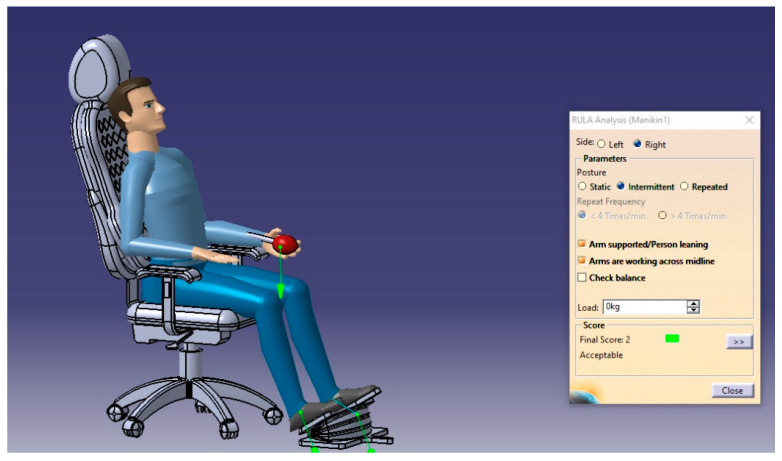
The RULA analysis output of the body’s right side in the intermittent posture.

**Figure 15:**
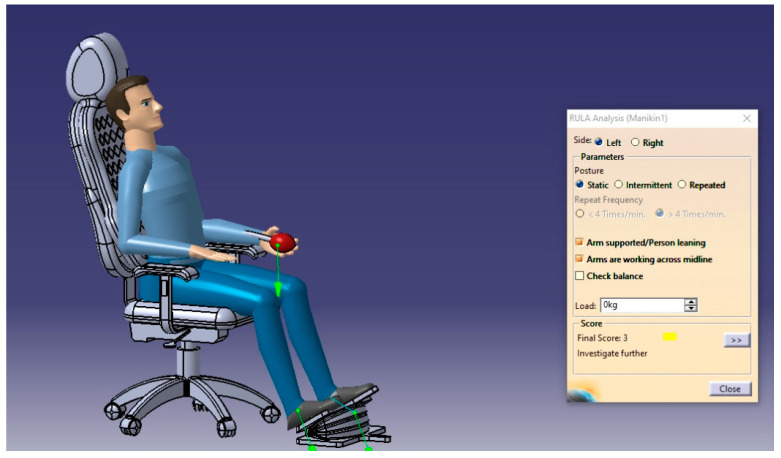
The RULA analysis output of the body’s left side in the static posture.

**Figure 16:**
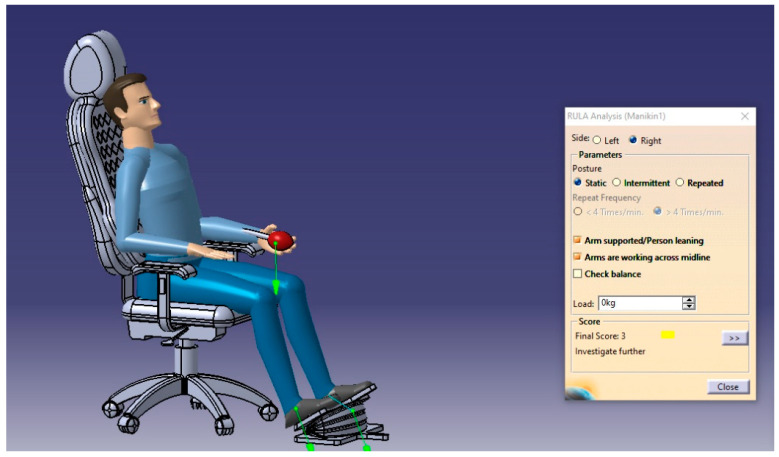
The RULA analysis output of the body’s right side in the static posture.

The RULA analysis focused on upper limb disorders. It assessed the posture of the neck, trunk, and upper limbs along with muscle functions and the external loads experienced by the body. Then, it presented the results as a risk score ranging from 1–7, where each value indicated a particular assessment of the sitting posture. For such analysis, two cases will be performed to check the sitting posture in the possible conditions (static sitting posture and intermittent sitting posture), and the results are presented in Figure 13, Figure 14, Figure 15 and Figure 16, respectively.

From the RULA index value shown in Table 18, the sitting postures of the user on the selected design concept (manual blowing system using the accordion blower) were acceptable and no adverse side effects can result where the RULA score does not exceed the value of 3, which means no risks and changing the sitting posture may be needed. 

Another type of ergonomic analysis can be performed in the CATIA software. This type of analysis was known as the “biomechanics single action analysis” which is used to provide biomechanical information in a certain sitting posture. This information includes the lumbar spinal loads, forces, and moments the manikin (user) experienced due to its sitting posture. 

**Figure 17:**
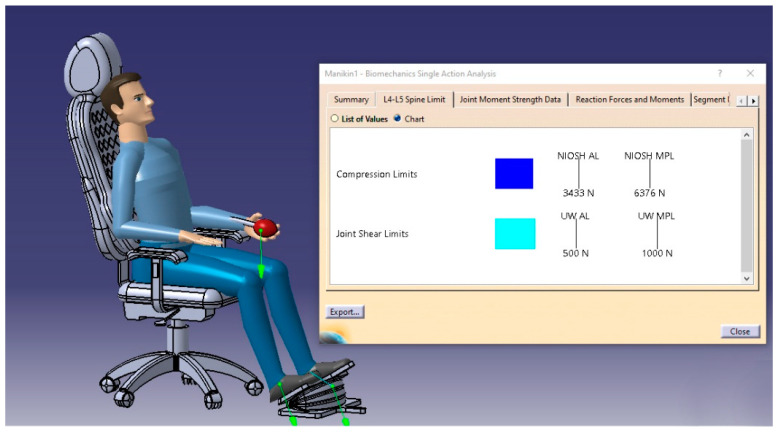
The L4_L5 spine limit.

The first of these analyses was the L4_L5 spine limit analysis, which introduces the two lowest vertebrae of the lumbar spine which are responsible for the trunk motion in multiple directions. The analysis was performed on CATIA software, and the results were shown in Figure 17.

#### 3.5.1. The L4_L5 Spine Limit

**Figure 18:**
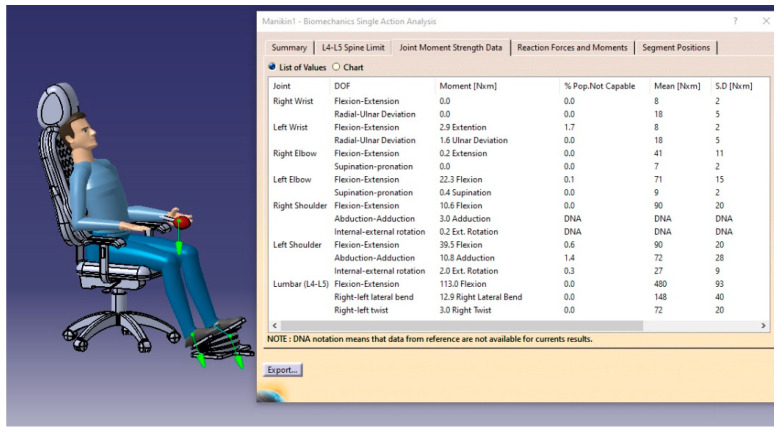
Joint moment strength data.

The findings of Figure 18 lead to the conclusion that neither the compression nor the joint shear limits were exceeded. The compression force value was 1511 N, which was below the 3433 N minimum permissible limit, indicating that it was an acceptable result and that there was no injury from the examined sitting posture. The National Institute for Occupational Safety and Health and the University of Waterloo set these criteria; nonetheless, the joint shear force was 245 N, less than the acceptable minimum permissible limit of 500 N.

#### 3.5.2. Joint Moment Strength Data

On the other hand, the CATIA software provides us with a thorough understanding of the user’s seated position. Figure 18 shows the value and direction of the moment for each body segment and statistical data on the user’s ability to feel moments on each segment of the body.

#### 3.5.3. Reaction Forces and Moments

**Figure 19:**
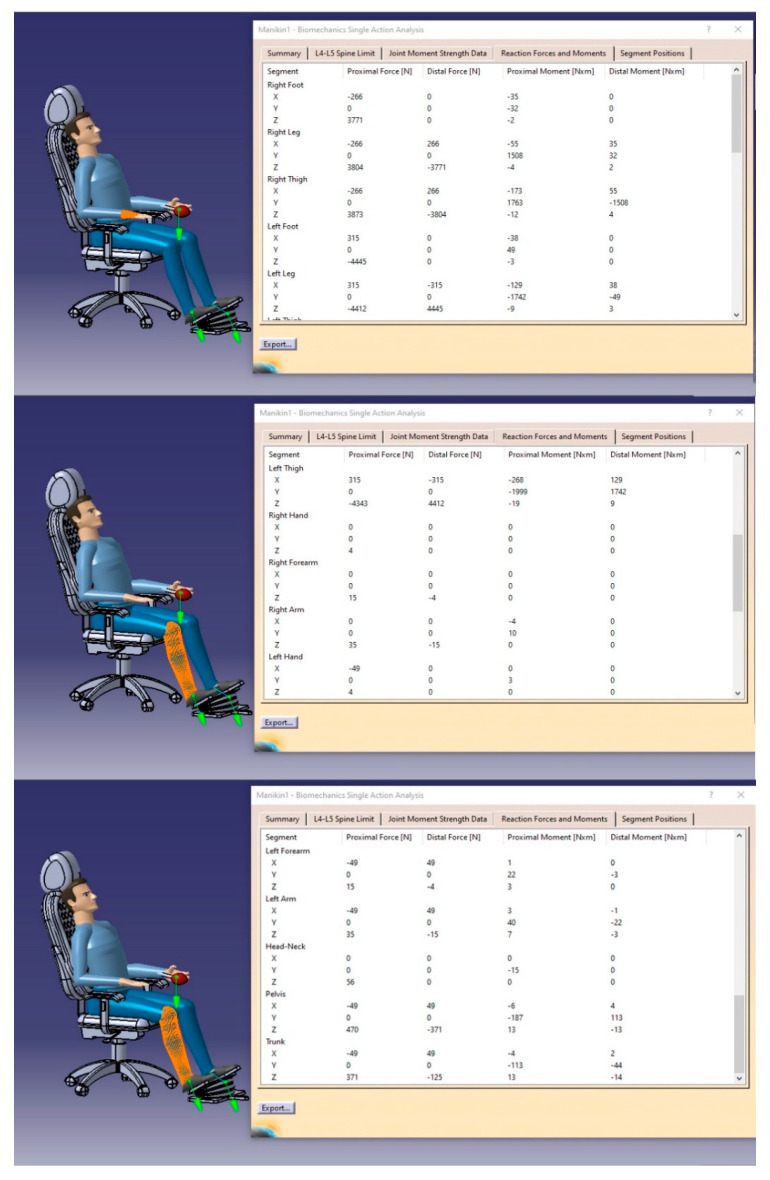
The data of the reaction forces and moments of the whole-body segments.

To satisfy the comprehensive view about the safety and health consideration of the user when using the proposed design of a seating cushion, CATIA software allows the user to perform an analysis that summarizes the reaction forces (N) and orthopedic moments (Nm) for the various human body segments, as demonstrated by Figure 19. The data is presented as a coordination number of the body segment and the values of the proximal and distal moments and reaction forces exerted on such body segments. 

## 4. Conclusions and Future Research

Prolonged sitting affects a human’s life negatively, where the effect is either physical or mental. Statistically, it was found that the risk of prolonged sitting is initiated after the person sits for four hours or longer in the same situation. In this study, a practical solution for enhancing safe prolonged sitting is developed as a chair attachment cushion that aims to instantaneously reduce the contact area between the chair and the occupants. Four hundred four participants were surveyed to confirm the need for such a product by proving the relation between lengthy sitting duration and skin symptoms such as skin sores, pressure ulcers, a numbness feeling, and the other side effects that resulted from the lengthy sitting situation. The cushion design was constructed based on the customer’s requirements and engineering characteristics. In the biomechanics single action study, the proposed design delivers an acceptable RULA index value for the tested sitting postures and works very safely. Thus, it can be used in different domains, where the users can do an exercise to perform the function of the cushion which can be reflected in extending the market of such products. For further development stages of the product life cycle, it is recommended that ergonomic analysis be performed experimentally on an actual physical product and conduct a performance study to make a comparison between the outcomes of the experimental data and the output of the ergonomic analysis simulation software, CATIA. 

## Figures and Tables

**Table 1:** A summary of the related research outcomes.

Research Study	Summary of the Outcomes of the Research
Mistarihi, M.Z. et al., 2020 [7]	Improving a wheelchair design that eliminates the awkward body postures of the disabled persons and their companions and reduces the effort exerted on the translation of the disabled persons from the wheelchair to other positions.
Davis et al., 2020 [8]	Highlighting the disadvantages of utilizing home goods for office work by conducting a quality improvement evaluation process. Because of this, using the home goods for extended work hours causes discomfort and unnatural posture.
Triglav, J. et al., 2019 [9]	Comparing standard and multi-axis chairs (core chairs). It was found that the latter delivers considerable improvement in the physiology and cognition of the chair users.
Ansari et al., 2018 [10]	Using anthropometric data of students during the educational process to provide an ergonomic chair design. The students’ muscle-skeletal abnormalities were minimized because of this proposed design.
Verver, M. et al., 2004 [11]	Utilizing the simulation technology to deal with the physical analysis of the sitting persons; using the finite element model (FEM) to determine the pressure distribution between the human and seat to improve an optimal vehicle seat.
Ali, A.Y., 2019 [12]	Using different tools to design and develop a multipurpose shoeshine chair. The techniques used in this study included the QFD approach, the concept development process, the concept selection process, and, in the end, cost analysis. The proposed model was competitive and succeeded in reducing the production cost of shoeshine chairs.
Schneider, L. et al., 2023 [13]	Providing a seat-integrated mobilization system that aims to reduce the muscle stiffness and discomfort of the truck driver by a dynamic mobilize system that results in a dynamic sitting posture which, prevents any negative impact of the excessive sitting.
Fan, J. et al., 2020 [14]	Utilizing the integration between the MCDM and FQFD to select an optimum design scheme in a cloudy environment and take the vehicle design as a case study.
Yuan, Y. and T. Guan., 2014 [15]	Involving the voice of the disabled person in the design of the manual wheelchair to improve its quality and maximize the user’s satisfaction through the AHP and KANO model.
Istifar, V. et al., 2021 [16]	Supplying a baby chair with a design that complies with Indonesian safety rules and takes ergonomics into account for the comfort of the infants using the chair.
Dorian STEF et al., 2022 [17]	A revolutionary dentist chair design is described that aims to reduce the chance of these disorders by separating the dental chair into two pillows that can be adjusted and inclined to reduce the risks of the MSDs of the dentist induced by prolonged sitting.
Ginting, R. et al., 2020 [18]	Applying the concurrent engineering approaches to modify the traditional wheelchair design. The goal was to help disabled and older people by modifying the wheelchair design to be with an adjustable lever and a bottle holder.
Otoda, Y. et al., 2018 [19]	Supplying a modified chair that will be used to assess the workers’ sitting posture, categorize their posture into various groups, and notify the person seated when to adjust their posture.
Daneshmandi H. et al., 2017 [20]	Four hundred forty-seven office workers were surveyed to determine the effects of prolonged sitting. As a result, it can be shown that the feeling of exhaustion will be maximized, performance decreased, blood pressure symptoms increased, and musculoskeletal disorders and awkward body postures for each of the knees, shoulders, thighs, and lower back of the workers increased.
Teng, T., 2020 [21]	Proposing a device (chair design) that is used to help people maintain a healthy sitting situation and keep them safer and comfortable.

**Table 2:** The characteristics of the four hundred four respondents for the research questionnaire.

Characteristic	Percentage				
Gender	Male: 49.3%			Female: 50.7%	
Length distribution	150–170 cm 48.3%	170–190 cm 23.5%	<150 cm 0.8%	150–190 cm 26.7%	>190 cm 0.7%
Weight distribution	<60 kg 5.9%	60–85 kg 72%	85–100 kg 10.1%	>100 kg 0.7%	>85 kg 11.3%
Avg. sitting hours	<3 h 8.7%	3–4 h 8.9%	4–5 h 12.1%	5–6 h 37.1%	>6 h 33.2%

**Table 3:** The characteristics of the expert’s team.

Field and Background	Size	Working Position
Mechanical engineering	21	Engineering workshop, AL Yarmouk University, Albalqa’a Applied University, Jordan University of Science and Technology
Physiotherapist	2	Special and governmental clinics
Biomedical engineering	5	AL Yarmouk University
Mechanical technician	11	Engineering workshop
Industrial engineering	10	Engineering workshop, AL Yarmouk University, Albalqa’a Applied University, Jordan University of Science and Technology, fabrication workshop

**Table 4:** Random index.

Size (n)	1	2	3	4	5	6	7	8	9
RI	0	0	0.58	0.9	1.12	1.24	1.32	1.41	1.45

**Table 5:** The average matrix of forty-nine decision makers.

Criteria	Design	Cost	Safety	Performance	Complexity	Expected Lifetime	Comfort Feeling	Assembly and Disassembly
Design	1	2	1	2	4	2	2	3
Cost	0.5	1	1	2	4	2	2	4
Safety	1	1	1	3	5	3	2	4
Performance	0.5	0.5	0.33	1	4	2	1	4
Complexity	0.25	0.25	0.2	0.25	1	1	1	1
Expected lifetime	0.5	0.5	0.33	0.5	1	1	1	3
Comfort feeling	0.5	0.5	0.5	1	1	1	1	5
Assembly and disassembly	0.33	0.25	0.25	0.25	1	0.33	0.2	1

**Table 6:** The average matrix of 49 experts uses fuzzy numbers.

Criteria	Design	Cost	Safety	Performance	Complexity	Expected Lifetime	Comfort Feeling	Assembly and Disassembly
Design	(1, 1, 1, 1)	(1, 3/2, 5/2, 3)	(1, 1, 1, 1)	(1, 3/2, 5/2, 3)	(3, 7/2, 9/2, 5)	(1, 3/2, 5/2, 3)	(1, 3/2, 5/2, 3)	(2, 5/2, 7/2, 4)
Cost	(1/3, 2/5, 2/3, 1)	(1, 1, 1, 1)	(1, 1, 1, 1)	(1, 3/2, 5/2, 3)	(3, 7/2, 9/2, 5)	(1, 3/2, 5/2, 3)	(1, 3/2, 5/2, 3)	(3, 7/2, 9/2, 5)
Safety	(1, 1, 1, 1)	(1, 1, 1, 1)	(1, 1, 1, 1)	(2, 5/2, 7/2, 4)	(4, 9/2, 11/2, 6)	(2, 5/2, 7/2, 4)	(1, 3/2, 5/2, 3)	(3, 7/2, 9/2, 5)
Performance	(1/3, 2/5, 2/3, 1)	(1/3, 2/5, 2/3, 1)	(1/4, 2/7, 2/5, 1/2)	(1, 1, 1, 1)	(3, 7/2, 9/2, 5)	(1, 3/2, 5/2, 3)	(1, 1, 1, 1)	(3, 7/2, 9/2, 5)
Complexity	(1/3, 2/5, 2/3, 1)	(1/5, 2/9, 2/7, 1/3)	(1/6, 2/11, 2/9, 1/4)	(1/5, 2/9, 2/7, 1/3)	(1, 1, 1, 1)	(1, 1, 1, 1)	(1, 1, 1, 1)	(1, 1, 1, 1)
Expected lifetime	(1/3, 2/5, 2/3, 1)	(1/3, 2/5, 2/3, 1)	(1/4, 2/7, 2/5, 1/2)	(1/3, 2/5, 2/3, 1)	(1, 1, 1, 1)	(1, 1, 1, 1)	(1, 1, 1, 1)	(2, 5/2, 7/2, 4)
Comfort feeling	(1/3, 2/5, 2/3, 1)	(1/3, 2/5, 2/3, 1)	(1/3, 2/5, 2/3, 1)	(1, 1, 1, 1)	(1, 1, 1, 1)	(1, 1, 1, 1)	(1, 1, 1, 1)	(4, 9/2, 11/2, 6)
Assembly and disassembly	(1/4, 2/7, 2/5, 1/2)	(1/5, 2/9, 2/7, 1/3)	(1/5, 2/9, 2/7, 1/3)	(1/5, 2/9, 2/7, 1/3)	(1, 1, 1, 1)	(1/4, 2/7, 2/5, 1/2)	(1/6, 2/11, 2/9, 1/4)	(1, 1, 1, 1)

**Table 7:** The coefficients of the criteria weight.

Criteria	$\alpha_{j}$	$\beta_{j}$	$\gamma_{j}$	$\delta_{j}$
Design	1.25	1.606	2.2316	2.518
Cost	1.147	1.42	1.95	2.258
Safety	1.622	1.867	2.29	2.482
Performance	0.841	0.978	1.316	1.573
Complexity	0.466	0.495	0.576	0.639
Expected lifetime	0.607	0.679	0.896	1.09
Comfort feeling	0.788	0.856	1.063	1.251
Assembly and disassembly	0.309	0.383	0.412	0.468

**Table 8:** The summations of α, β, γ, δ.

$\alpha_{j}$	$\beta_{j}$	$\gamma_{j}$	$\delta_{j}$
7.03	8.284	10.7346	12.279

**Table 9:** The inverse of the summation of the coefficients of the criteria weight.

$\alpha^{-1}$	$\beta^{-1}$	$\gamma^{-1}$	$\delta^{-1}$
0.142248	0.120715	0.093157	0.08144

**Table 10:** The weights of the criteria using the FAHP approach.

Weights of the Criteria	Weights				Defuzzification
Design	0.102	0.15	0.269	0.358	0.216328656
Cost	0.093	0.132	0.235	0.321	0.191659751
Safety	0.132	0.174	0.276	0.353	0.230978988
Performance	0.068	0.091	0.159	0.224	0.13203028
Complexity	0.038	0.046	0.07	0.091	0.060022588
Expected lifetime	0.049	0.063	0.108	0.155	0.091218535
Comfort feeling	0.064	0.08	0.128	0.178	0.109708305
Assembly and disassembly	0.025	0.036	0.05	0.067	0.043760607

**Table 11:** The weights of eight criteria using the fuzzy TOPSIS.

Criteria	Weights
Design	(1, 5.9, 6.9, 10)
Cost	(1, 7.3, 8.3, 10)
Safety	(3, 7, 8, 10)
Performance	(0, 6.5, 7.5, 10)
Complexity	(0, 3.3, 4.3, 10)
Expected lifetime	(0, 5.7, 6.7, 10)
Comfort feeling	(1, 7, 8, 10)
Assembly and disassembly	(0, 3.7, 4.7, 10)

**Table 12:** The final ranking of four design concepts using the fuzzy AHP.

Alternatives	Fuzzy Values	Defuzzification	Final Ranking
Auto motor	3.574002, 4.571164,5.69736, 6.731633	5.140448	2
Manual pedal	3.825977, 4.630089, 5.446553, 6.195986	5.029208	3
Auto gas	2.319633, 3.321693, 4.329336, 5.345397	3.827848	4
Manual accordion	5.804282, 6.827985, 7.858514, 8.852295	7.338262	1

**Table 13:** The final ranking of four design concepts using the fuzzy TOPSIS.

Alternatives	Fuzzy Values	Defuzzification	Final Ranking
Auto motor	3.544218, 4.588098, 5.728204, 6.737245	5.152345	2
Manual pedal	4.210885, 4.53158, 5.406062, 6.270408	5.05943	3
Auto gas	1.897959, 3.462702, 4.47419, 5.540816	3.885427	4
Manual accordion	6.170068, 6.632301, 7.602716, 8.431122	7.178537	1

**Table 14:** The results of the sensitivity analysis of the fuzzy AHP.

Experiment Number	Weights	Z Values				Rank
		Z1	Z2	Z3	Z4	
E1	Safety: 0.132, 0.174, 0.276, 0.353 Design: 0.102, 0.15, 0.269, 0.358	5.207262	6.645305	3.233359	7.578954	Z4 > Z2 > Z1 > Z3
E2	Safety: 0.132, 0.174, 0.276, 0.353 Cost: 0.093, 0.132, 0.235, 0.321	4.816943	3.788485	3.673686	8.069864	Z4 > Z1 > Z2 > Z3
E3	Safety: 0.132, 0.174, 0.276, 0.353 Performance: 0.068, 0.091, 0.159, 0.224	5.238485	6.619165	3.316781	7.622298	Z4 > Z2 > Z1 > Z3
E4	Cost: 0.093, 0.132, 0.235, 0.321 Design: 0.102, 0.15, 0.269, 0.358	4.954516	3.394195	3.958909	7.926444	Z4 > Z1 > Z3 > Z2
E5	Performance: 0.068, 0.091, 0.159, 0.224 Design: 0.102, 0.15, 0.269, 0.358	5.414504	6.313091	3.627136	7.426133	Z4 > Z2 > Z1 > Z3
E6	Performance: 0.068, 0.091, 0.159, 0.224 Cost: 0.093, 0.132, 0.235, 0.321	4.913327	2.524053	4.240969	8.065496	Z4 > Z1 > Z3 > Z2

**Table 15:** The results of the sensitivity analysis of the fuzzy TOPSIS.

Experiment Number	Weights	Z Values				Rank
		Z1	Z2	Z3	Z4	
E1	Safety: 3, 7, 8, 10 Cost: 1, 7.3, 8.3, 10	4.805773	3.61245	3.702806	8.085023	Z4 > Z1 > Z3 > Z2
E2	Safety: 3, 7, 8, 10 Comfort feeling: 3, 7, 8, 10	5.127551	6.390306	3.178571	7.359694	Z4 > Z2 > Z1 > Z3
E3	Safety: 3, 7, 8, 10 Performance: 0, 6.5, 7.5, 10	5.296713	6.586245	3.357448	7.608742	Z4 > Z2 > Z1 > Z3
E4	Cost: 1, 7.3, 8.3, 10 Comfort feeling: 1, 7, 8, 10	4.842384	2.875235	3.946143	7.7246	Z4 > Z1 > Z3 > Z2
E5	Performance: 0, 6.5, 7.5, 10 Cost: 1, 7.3, 8.3, 10	4.95058	2.586146	4.247846	8.077032	Z4 > Z1 > Z3 > Z2
E6	Performance: 0, 6.5, 7.5, 10 Comfort feeling: 1, 7, 8, 10	5.369556	5.967082	3.575976	7.135264	Z4 > Z2 > Z1 > Z3

**Table 16:** Summary of customers’ preferences using HoQ.

				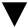		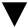	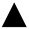	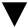	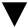
	%	Weight Out of 5	Engineering Characteristics 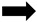	Total Cost EC1		Design Complexity EC2	Type of Material EC3	Method of Production (Production Complexity) EC5	Air cell Puffing Time EC6
			Customer Requirement 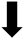	Cost of Production EC11	Cost of Equipment EC13				
	13.3%	4	Price CR1	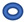	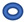		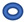	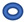	
	14%	4.2	Safety CR2	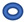	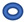	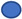	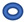	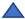	
	10.7%	3.2	Appearance and aesthetic features CR3	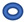	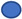	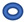	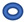	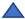	
	12.7%	3.8	Ease of (use, assembly, and disassembly) CR4	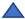	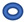	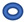	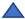	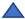	
	12.3%	3.7	Ease of repair CR5		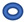	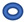	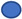	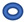	
	11.3%	3.4	Ease of cleaning CR6			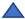	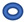		
	14%	4.2	Performance CR7		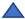	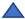			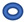
	11.3%	3.4	No conflicting with the main functionality of the chair CR8	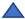	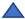	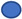			
Total	100%	30		366	528.1	422.5	493.3	267.8	126
	Ratio			16.8%	24.2%	19.4%	22.6%	12.3%	5.71%
	Rank			4	1	3	2	5	6

**Table 17:** The legend of the HoQ.

Symbol	Meaning	Weight
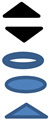	The less the better	N/A
	The more the better	N/A
	Strong relationship matrix	9
	Medium relationship matrix	3
	Weak relationship matrix	1
	No relationship matrix	0

**Table 18:** RULA score index and the certain process for each score value.

RULA Score	Meaning
1–2	Negligible risk, no action required
3–4	Low risk, change may be needed
5–6	Medium risk, further investigation, change soon
6+	Very high risk, implement change now

## Data Availability

The datasets generated during and/or analyzed during the current study are available from the corresponding author on reasonable request.

## Appendix A

**Table A1:** The evaluation of the criteria fuzzy TOPSIS approach.

Criteria	Design	Cost	Safety	Performance	Complexity	Expected Lifetime	Comfort Feeling	Assembly and Disassembly
DM * 1	moderate	very good	very good	very good	moderate	good	very good	poor
DM 2	good	good	very good	good	poor	good	very good	good
DM 3	very good	good	good	moderate	moderate	good	very good	very good
DM 4	poor	very good	very good	good	very poor	moderate	good	very good
DM 5	very good	very good	very good	good	poor	very good	very good	poor
DM 6	very good	very good	very good	good	moderate	very poor	very good	poor
DM 7	good	very good	good	very good	poor	good	good	very poor
DM 8	very good	very good	very good	good	poor	very good	moderate	poor
DM 9	moderate	very good	good	good	very poor	poor	very good	very good
DM 10	moderate	very good	very good	good	poor	poor	good	poor
DM 11	good	good	very good	very good	good	good	very good	good
DM 12	very good	very good	very good	good	very poor	moderate	good	good
DM 13	good	very good	good	very good	poor	very good	very good	very poor
DM 14	poor	very good	good	very poor	poor	very good	very good	moderate
DM 15	moderate	very good	good	moderate	good	very good	good	good
DM 16	very good	good	very good	moderate	good	very poor	poor	good
DM 17	very good	good	very good	very good	very poor	very good	good	very poor
DM 18	very good	good	very good	good	moderate	very good	very good	good
DM 19	good	good	good	good	good	good	very good	good
DM 20	very good	poor	good	good	good	good	good	very poor
DM 21	very good	very good	very good	very good	very good	very good	very good	very good
DM 22	very good	very good	very good	very good	poor	very good	very good	poor
DM 23	good	good	good	good	good	good	good	good
DM 24	moderate	very good	very good	good	poor	good	very good	good
DM 25	good	very good	good	very good	poor	moderate	good	very poor
DM 26	very good	very good	very good	very good	moderate	good	very good	very poor
DM 27	very good	very good	very good	good	poor	moderate	moderate	very good
DM 28	good	very good	very good	good	poor	moderate	good	very poor
DM 29	poor	very good	good	good	very poor	very good	very good	poor
DM 30	good	very good	good	very good	poor	moderate	good	very poor
DM 31	good	very good	good	very good	poor	good	good	very poor
DM 32	very good	moderate	moderate	good	very poor	very good	very good	moderate
DM 33	moderate	very good	very good	very good	good	very good	very good	poor
DM 34	good	very good	very good	poor	good	good	good	poor
DM 35	good	very good	very good	good	very poor	moderate	good	very poor
DM 36	moderate	very good	very good	good	very poor	good	good	very poor
DM 37	very poor	very good	good	good	good	good	very good	moderate
DM 38	moderate	very good	good	very good	poor	good	very good	poor
DM 39	good	very good	good	very good	good	good	good	very poor
DM 40	moderate	very good	very good	good	poor	poor	good	very poor
DM 41	very good	good	very good	good	good	good	very good	good
DM 42	good	very good	very good	very good	poor	good	very good	poor
DM 43	very good	very good	good	very good	poor	very good	very good	very poor
DM 44	moderate	very good	very good	good	moderate	moderate	good	moderate
DM 45	good	very good	moderate	very good	very poor	poor	good	good
DM 46	very good	very good	very good	very good	good	very good	very good	good
DM 47	good	good	very good	very good	good	very good	very good	very good
DM 48	very good	good	good	moderate	moderate	good	very good	good
DM 49	poor	very good	moderate	very good	poor	moderate	very good	very poor

* DM stands for decision makers.

**Table A2:** The evaluation of the four proposed designs.

A. The Evaluation of the Automatic Blowing System Using the Driving motor						
The Automatic Blowing System Using the Driving Motor						
	Goodness level	Very poor	Poor	Moderate	Good	Very good
Criteria						
Design		0	0	35	7	7
Cost		0	0	49	0	0
Safety		0	3	33	9	4
Performance		1	1	37	6	4
Complexity		6	11	14	9	9
Expected lifetime		1	6	14	15	13
Comfort feeling		0	2	35	5	7
Assembly and disassembly		7	8	11	17	6
**B. The evaluation of the manual blowing system using the pedaling mechanism**						
**The manual blowing system using the pedaling mechanism**						
	Goodness level	Very poor	Poor	Moderate	Good	Very good
Criteria						
Design		0	3	5	34	7
Cost		49	0	0	0	0
Safety		0	0	5	30	14
Performance		0	0	10	37	2
Complexity		9	8	18	9	5
Expected lifetime		3	9	8	16	13
Comfort feeling		1	6	4	36	2
Assembly and disassembly		10	8	11	19	1
**C. The evaluation of the automatic blowing system using the gas expanding principle**						
**The automatic blowing system using the gas expanding principle**						
	Goodness level	Very poor	Poor	Moderate	Good	Very good
Criteria						
Design		2	33	4	9	1
Cost		0	0	49	0	0
Safety		15	23	4	5	2
Performance		3	26	7	11	2
Complexity		5	10	14	9	5
Expected lifetime		0	12	14	19	4
Comfort feeling		9	27	4	5	4
Assembly and disassembly		8	7	15	15	4
**D. The evaluation of the manual blowing system using the accordion blower**						
**The manual blowing system using the accordion blower**						
	Goodness level	Very poor	Poor	Moderate	Good	Very good
Criteria						
Design		1	0	9	5	34
Cost		0	0	0	0	49
Safety		0	0	2	15	32
Performance		2	0	3	13	31
Complexity		2	6	12	18	11
Expected lifetime		1	4	15	16	13
Comfort feeling		1	3	7	12	26
Assembly and disassembly		2	10	10	12	15

**Table A3:** Criteria evaluation matrix using the fuzzy numbers.

Criteria	Design	Cost	Safety	Performance	Complexity	Expected Lifetime	Comfort Feeling	Assembly and Disassembly
DM * 1	(3, 4, 5, 6)	(7, 8, 9, 10)	(7, 8, 9, 10)	(7, 8, 9, 10)	(3, 4, 5, 6)	(5, 6, 7, 8)	(7, 8, 9, 10)	(1, 2, 3, 4)
DM 2	(5, 6, 7, 8)	(5, 6, 7, 8)	(7, 8, 9, 10)	(5, 6, 7, 8)	(1, 2, 3, 4)	(5, 6, 7, 8)	(7, 8, 9, 10)	(5, 6, 7, 8)
DM 3	(7, 8, 9, 10)	(5, 6, 7, 8)	(5, 6, 7, 8)	(3, 4, 5, 6)	(3, 4, 5, 6)	(5, 6, 7, 8)	(7, 8, 9, 10)	(7, 8, 9, 10)
DM 4	(1, 2, 3, 4)	(7, 8, 9, 10)	(7, 8, 9, 10)	(5, 6, 7, 8)	(0, 1, 2, 3)	(3, 4, 5, 6)	(5, 6, 7, 8)	(7, 8, 9, 10)
DM 5	(7, 8, 9, 10)	(7, 8, 9, 10)	(7, 8, 9, 10)	(5, 6, 7, 8)	(1, 2, 3, 4)	(7, 8, 9, 10)	(7, 8, 9, 10)	(1, 2, 3, 4)
DM 6	(7, 8, 9, 10)	(7, 8, 9, 10)	(7, 8, 9, 10)	(5, 6, 7, 8)	(3, 4, 5, 6)	(0, 1, 2, 3)	(7, 8, 9, 10)	(1, 2, 3, 4)
DM 7	(5, 6, 7, 8)	(7, 8, 9, 10)	(5, 6, 7, 8)	(7, 8, 9, 10)	(1, 2, 3, 4)	(5, 6, 7, 8)	(5, 6, 7, 8)	(0, 1, 2, 3)
DM 8	(7, 8, 9, 10)	(7, 8, 9, 10)	(7, 8, 9, 10)	(5, 6, 7, 8)	(1, 2, 3, 4)	(7, 8, 9, 10)	(3, 4, 5, 6)	(1, 2, 3, 4)
DM 9	(3, 4, 5, 6)	(7, 8, 9, 10)	(5, 6, 7, 8)	(5, 6, 7, 8)	(0, 1, 2, 3)	(1, 2, 3, 4)	(7, 8, 9, 10)	(7, 8, 9, 10)
DM 10	(3, 4, 5, 6)	(7, 8, 9, 10)	(7, 8, 9, 10)	(5, 6, 7, 8)	(1, 2, 3, 4)	(1, 2, 3, 4)	(5, 6, 7, 8)	(1, 2, 3, 4)
DM 11	(5, 6, 7, 8)	(5, 6, 7, 8)	(7, 8, 9, 10)	(7, 8, 9, 10)	(5, 6, 7, 8)	(5, 6, 7, 8)	(7, 8, 9, 10)	(5, 6, 7, 8)
DM 12	(7, 8, 9, 10)	(7, 8, 9, 10)	(7, 8, 9, 10)	(5, 6, 7, 8)	(0, 1, 2, 3)	(3, 4, 5, 6)	(5, 6, 7, 8)	(5, 6, 7, 8)
DM 13	(5, 6, 7, 8)	(7, 8, 9, 10)	(5, 6, 7, 8)	(7, 8, 9, 10)	(1, 2, 3, 4)	(7, 8, 9, 10)	(7, 8, 9, 10)	(0, 1, 2, 3)
DM 14	(1, 2, 3, 4)	(7, 8, 9, 10)	(5, 6, 7, 8)	(0, 1, 2, 3)	(1, 2, 3, 4)	(7, 8, 9, 10)	(7, 8, 9, 10)	(3, 4, 5, 6)
DM 15	(3, 4, 5, 6)	(7, 8, 9, 10)	(5, 6, 7, 8)	(3, 4, 5, 6)	(5, 6, 7, 8)	(7, 8, 9, 10)	(5, 6, 7, 8)	(5, 6, 7, 8)
DM 16	(7, 8, 9, 10)	(5, 6, 7, 8)	(7, 8, 9, 10)	(3, 4, 5, 6)	(5, 6, 7, 8)	(0, 1, 2, 3)	(1, 2, 3, 4)	(5, 6, 7, 8)
DM 17	(7, 8, 9, 10)	(5, 6, 7, 8)	(7, 8, 9, 10)	(7, 8, 9, 10)	(0, 1, 2, 3)	(7, 8, 9, 10)	(5, 6, 7, 8)	(0, 1, 2, 3)
DM 18	(7, 8, 9, 10)	(5, 6, 7, 8)	(7, 8, 9, 10)	(5, 6, 7, 8)	(3, 4, 5, 6)	(7, 8, 9, 10)	(7, 8, 9, 10)	(5, 6, 7, 8)
DM 19	(5, 6, 7, 8)	(5, 6, 7, 8)	(5, 6, 7, 8)	(5, 6, 7, 8)	(5, 6, 7, 8)	(5, 6, 7, 8)	(7, 8, 9, 10)	(5, 6, 7, 8)
DM 20	(7, 8, 9, 10)	(1, 2, 3, 4)	(5, 6, 7, 8)	(5, 6, 7, 8)	(5, 6, 7, 8)	(5, 6, 7, 8)	(5, 6, 7, 8)	(0, 1, 2, 3)
DM 21	(7, 8, 9, 10)	(7, 8, 9, 10)	(7, 8, 9, 10)	(7, 8, 9, 10)	(7, 8, 9, 10)	(7, 8, 9, 10)	(7, 8, 9, 10)	(7, 8, 9, 10)
DM 22	(7, 8, 9, 10)	(7, 8, 9, 10)	(7, 8, 9, 10)	(7, 8, 9, 10)	(1, 2, 3, 4)	(7, 8, 9, 10)	(7, 8, 9, 10)	(1, 2, 3, 4)
DM 23	(5, 6, 7, 8)	(5, 6, 7, 8)	(5, 6, 7, 8)	(5, 6, 7, 8)	(5, 6, 7, 8)	(5, 6, 7, 8)	(5, 6, 7, 8)	(5, 6, 7, 8)
DM 24	(3, 4, 5, 6)	(7, 8, 9, 10)	(7, 8, 9, 10)	(5, 6, 7, 8)	(1, 2, 3, 4)	(5, 6, 7, 8)	(7, 8, 9, 10)	(5, 6, 7, 8)
DM 25	(5, 6, 7, 8)	(7, 8, 9, 10)	(5, 6, 7, 8)	(7, 8, 9, 10)	(1, 2, 3, 4)	(3, 4, 5, 6)	(5, 6, 7, 8)	(0, 1, 2, 3)
DM 26	(7, 8, 9, 10)	(7, 8, 9, 10)	(7, 8, 9, 10)	(7, 8, 9, 10)	(3, 4, 5, 6)	(5, 6, 7, 8)	(7, 8, 9, 10)	(0, 1, 2, 3)
DM 27	(7, 8, 9, 10)	(7, 8, 9, 10)	(7, 8, 9, 10)	(5, 6, 7, 8)	(1, 2, 3, 4)	(3, 4, 5, 6)	(3, 4, 5, 6)	(7, 8, 9, 10)
DM 28	(5, 6, 7, 8)	(7, 8, 9, 10)	(7, 8, 9, 10)	(5, 6, 7, 8)	(1, 2, 3, 4)	(3, 4, 5, 6)	(5, 6, 7, 8)	(0, 1, 2, 3)
DM 29	(1, 2, 3, 4)	(7, 8, 9, 10)	(5, 6, 7, 8)	(5, 6, 7, 8)	(0, 1, 2, 3)	(7, 8, 9, 10)	(7, 8, 9, 10)	(1, 2, 3, 4)
DM 30	(5, 6, 7, 8)	(7, 8, 9, 10)	(5, 6, 7, 8)	(7, 8, 9, 10)	(1, 2, 3, 4)	(3, 4, 5, 6)	(5, 6, 7, 8)	(0, 1, 2, 3)
DM 31	(5, 6, 7, 8)	(7, 8, 9, 10)	(5, 6, 7, 8)	(7, 8, 9, 10)	(1, 2, 3, 4)	(5, 6, 7, 8)	(5, 6, 7, 8)	(0, 1, 2, 3)
DM 32	(7, 8, 9, 10)	(3, 4, 5, 6)	(3, 4, 5, 6)	(5, 6, 7, 8)	(0, 1, 2, 3)	(7, 8, 9, 10)	(7, 8, 9, 10)	(3, 4, 5, 6)
DM 33	(3, 4, 5, 6)	(7, 8, 9, 10)	(7, 8, 9, 10)	(7, 8, 9, 10)	(5, 6, 7, 8)	(7, 8, 9, 10)	(7, 8, 9, 10)	(1, 2, 3, 4)
DM 34	(5, 6, 7, 8)	(7, 8, 9, 10)	(7, 8, 9, 10)	(1, 2, 3, 4)	(5, 6, 7, 8)	(5, 6, 7, 8)	(5, 6, 7, 8)	(1, 2, 3, 4)
DM 35	(5, 6, 7, 8)	(7, 8, 9, 10)	(7, 8, 9, 10)	(5, 6, 7, 8)	(0, 1, 2, 3)	(3, 4, 5, 6)	(5, 6, 7, 8)	(0, 1, 2, 3)
DM 36	(3, 4, 5, 6)	(7, 8, 9, 10)	(7, 8, 9, 10)	(5, 6, 7, 8)	(0, 1, 2, 3)	(5, 6, 7, 8)	(5, 6, 7, 8)	(0, 1, 2, 3)
DM 37	(0, 1, 2, 3)	(7, 8, 9, 10)	(5, 6, 7, 8)	(5, 6, 7, 8)	(5, 6, 7, 8)	(5, 6, 7, 8)	(7, 8, 9, 10)	(3, 4, 5, 6)
DM 38	(3, 4, 5, 6)	(7, 8, 9, 10)	(5, 6, 7, 8)	(7, 8, 9, 10)	(1, 2, 3, 4)	(5, 6, 7, 8)	(7, 8, 9, 10)	(1, 2, 3, 4)
DM 39	(5, 6, 7, 8)	(7, 8, 9, 10)	(5, 6, 7, 8)	(7, 8, 9, 10)	(5, 6, 7, 8)	(5, 6, 7, 8)	(5, 6, 7, 8)	(0, 1, 2, 3)
DM 40	(3, 4, 5, 6)	(7, 8, 9, 10)	(7, 8, 9, 10)	(5, 6, 7, 8)	(1, 2, 3, 4)	(1, 2, 3, 4)	(5, 6, 7, 8)	(0, 1, 2, 3)
DM 41	(7, 8, 9, 10)	(5, 6, 7, 8)	(7, 8, 9, 10)	(5, 6, 7, 8)	(5, 6, 7, 8)	(5, 6, 7, 8)	(7, 8, 9, 10)	(5, 6, 7, 8)
DM 42	(5, 6, 7, 8)	(7, 8, 9, 10)	(7, 8, 9, 10)	(7, 8, 9, 10)	(1, 2, 3, 4)	(5, 6, 7, 8)	(7, 8, 9, 10)	(1, 2, 3, 4)
DM 43	(7, 8, 9, 10)	(7, 8, 9, 10)	(5, 6, 7, 8)	(7, 8, 9, 10)	(1, 2, 3, 4)	(7, 8, 9, 10)	(7, 8, 9, 10)	(0, 1, 2, 3)
DM 44	(3, 4, 5, 6)	(7, 8, 9, 10)	(7, 8, 9, 10)	(5, 6, 7, 8)	(3, 4, 5, 6)	(3, 4, 5, 6)	(5, 6, 7, 8)	(3, 4, 5, 6)
DM 45	(5, 6, 7, 8)	(7, 8, 9, 10)	(3, 4, 5, 6)	(7, 8, 9, 10)	(0, 1, 2, 3)	(1, 2, 3, 4)	(5, 6, 7, 8)	(5, 6, 7, 8)
DM 46	(7, 8, 9, 10)	(7, 8, 9, 10)	(7, 8, 9, 10)	(7, 8, 9, 10)	(5, 6, 7, 8)	(7, 8, 9, 10)	(7, 8, 9, 10)	(5, 6, 7, 8)
DM 47	(5, 6, 7, 8)	(5, 6, 7, 8)	(7, 8, 9, 10)	(7, 8, 9, 10)	(5, 6, 7, 8)	(7, 8, 9, 10)	(7, 8, 9, 10)	(7, 8, 9, 10)
DM 48	(7, 8, 9, 10)	(5, 6, 7, 8)	(5, 6, 7, 8)	(3, 4, 5, 6)	(3, 4, 5, 6)	(5, 6, 7, 8)	(7, 8, 9, 10)	(5, 6, 7, 8)
DM 49	(1, 2, 3, 4)	(7, 8, 9, 10)	(3, 4, 5, 6)	(7, 8, 9, 10)	(1, 2, 3, 4)	(3, 4, 5, 6)	(7, 8, 9, 10)	(0, 1, 2, 3)

* DM stands for decision maker.
